# Diallyl disulfide alleviates hypercholesterolemia induced by a western diet by suppressing endoplasmic reticulum stress in apolipoprotein E-deficient mice

**DOI:** 10.1186/s12906-023-03920-1

**Published:** 2023-05-03

**Authors:** Hyun Ju Kim, Mijeong Kim

**Affiliations:** 1 Kimchi Functionality Research Group, World Institute of Kimchi, Nam-Gu, Gwangju, 61755 Republic of Korea; 2 Department of Food Science and Nutrition and Kimchi Research Institute, Pusan National University, 2, Busandaehak-ro 63beon-gil, Geumjeong-gu, Busan, 46241 Republic of Korea

**Keywords:** Endoplasmic reticulum stress, Diallyl disulfide, Leptin, Hypercholesterolemia, Apolipoprotein E deficient mice

## Abstract

**Background:**

The endoplasmic reticulum (ER) plays a pivotal role in maintaining cellular metabolic homeostasis. ER stress refers to the accumulation of misfolded proteins, which can trigger an unfolded protein response for survival or death in the cells. Diallyl disulfide (DADS), a major active compound in garlic, has many health benefits for patients with metabolic diseases, especially cardiovascular or fatty liver diseases. However, its role in attenuating hypercholesterolemia by suppressing ER stress remains unknown. Therefore, in this study, we determined whether DADS supplementation could reduce ER stress in apolipoprotein E-deficient (ApoE^−/−^) mice fed a Western-type diet (WD).

**Methods:**

ApoE^−/−^ mice were fed either a WD alone or a WD supplemented with 0.1% DADS for 12 weeks (*n* = 10). Levels of plasma total cholesterol, triglyceride, leptin, and insulin were determined. Western blotting was performed to measure protein levels involved in ER stress markers. Histology and Immunostaining were performed on aortic root sections to confirm the effect of DADS on histology and expression of ER chaperone protein GRP78.

**Results:**

The metabolic parameters showed that increases in fat weight, leptin resistance, and hypercholesterolemia were reversed in DADS-supplemented mice (*p* < 0.05). In addition, DADS ameliorated not only the protein of ER stress markers, phospho-eukaryotic initiation factor 2 subunit alpha and C/EBP homologous protein in the liver (*p* < 0.05) but also glucose-related protein 78 localization in the aorta.

**Conclusions:**

This indicates that DADS inhibits diet-induced hypercholesterolemia, at least in parts by regulating ER stress markers. DADS may be a good candidate for treating individuals with diet-induced hypercholesterolemia.

**Supplementary Information:**

The online version contains supplementary material available at 10.1186/s12906-023-03920-1.

## **Background**

The endoplasmic reticulum (ER) is a pivotal site that mediates protein folding, maturation, quality control, and trafficking. Under conditions of overnutrition, ER homeostasis is disturbed by the overload of unfolded proteins and calcium depletion, which is referred to as ER stress. Dysfunction of the ER is associated with numerous metabolic disorders, including obesity, diabetes, hypercholesterolemia, and atherosclerosis [1]. High levels of free cholesterol (FC) in macrophage foam cells and the formation of free radicals by activated vascular and inflammatory cells are considered to be critical processes in the development of atherosclerotic lesions and can trigger ER stress [2, 3]. The accumulation of unfolded proteins by oxidized low-density lipoproteins (oxLDL) in the ER induces unfolded protein response (UPR) in human endothelial cells, which is characterized by the stimulation of ER stress pathways. This includes induction of eukaryotic initiation factor 2 subunit alpha phosphorylation and expression of X-box binding protein (XBP) and C/EBP homologous protein (CHOP), resulting in apoptotic cell death and disruption of protein folding [4]. The 78-kD glucose-regulated/binding immunoglobulin protein (GRP78/BiP), a major ER molecular chaperone, is vital for regulating ER stress by promoting the correct folding and assembly of proteins. Upon ER stress, GRP78/BiP anchors misfolded proteins, cause activation of ER stress sensors and UPR inauguration. This results in stabilization of protein folding, which protects cells from prolonged or severe ER stress [5, 6]. ER stress is also implicated in leptin resistance, and activation of the UPR can block the leptin signaling network and contribute to energy imbalances in obesity and related diseases [7].

Despite remarkable advances in cardiovascular disease, atherosclerosis ranks a major cause of death worldwide. Atherosclerosis refers to the accumulation of lipid-rich plaques in large arteries [8]. Accumulation of lipids and local inflammatory elements in endothelial cells, increased infiltration of macrophages, and migration of smooth muscle cells are hallmarks of atherosclerotic plaque progression [9]. Excess FC and oxLDL entry and retention in macrophages leads to the accumulation of cholesterol, triggering the UPR, which occurs in all stages of atherosclerotic lesions [5]. Although ER stress is implicated in the rupture and thrombosis of atherosclerotic plaques, UPR is regarded as a new adaptive response to cell survival [1].

Diallyl disulfide (DADS), a potent compound of garlic, has anti-inflammatory, antioxidant, antimicrobial, detoxifying, protective for cardiovascular and neurological systems, anticancer, and regulatory for metabolic systems effects [10–12]. Recently, garlic and its major components have been implicated in the prevention of metabolic and vascular dysfunction by enhancing thermogenesis and inhibiting mitochondrial oxidative stress in obesity [13–15]. However, it is still unclear whether dietary supplementation with DADS can attenuate hypercholesterolemia and slow the progression of atherosclerotic lesions by reducing ER stress. To address these questions, we examined whether dietary DADS could attenuate hypercholesterolemia-induced ER stress.

## Materials and methods

### Materials

DADS was purchased from Sigma Aldrich (St. Louis, MO, USA).

### Animals and diets

All procedures were performed in accordance with guidelines approved by the Institutional Animal Care and Use Committee of the Korea Food Research Institute (KFRI-M-12,030). Six-week-old ApoE^−/−^ (male, 20 ~ 25 g) and wild type (WT, male, 20 ~ 23 g) mice were obtained from Jackson Laboratories (Bar Harbor, ME, USA) and maintained under a light-dark (12/12-h) cycle and temperatures of 21–23 °C. WT mice were fed a normal chow diet (Research Diet Inc., New Brunswick, NJ, USA) and ApoE^−/−^ mice were assigned one of three experimental diets: WD (60 kcal from fat, *n* = 10); Research Diet; or WD + 0.1% DADS (*n* = 10). The compositions of the experimental diets are presented in Table 1. Body weight and food intake were recorded once per week and every other day, respectively. At 12 weeks, the mice were fasted overnight and sacrificed by CO_2_ asphyxiation. The blood were collected from cardiac puncture and stored in a tube treated heparin. After centrifugation1,200×g, 10 min, room temperature, plasma was aliquoted and stored at -80 °C. The organs were isolated and stored at -80 °C until use or in 10% buffered neutral formalin for histological analysis.

**Table 1:** Diet composition (g/kg diet)

		ApoE^−/−^ + WD			
		WD	0.1% DADS		
Casein		195	195		
DL-methionine		3	3		
Corn starch		50	50		
Maltodextrin 10		100	100		
Sucrose		341	341		
Cellulose, BW200		50	50		
Milk fat, Anhydrous		200	200		
Corn oil		10	10		
Mineral mix		35	35		
Calcium carbonate		4	4		
Vitamin mix		10	10		
Choline bitartrate		2	2		
Cholesterol, USP		1.5	1.5		
Ethoxyquin		0.04	0.04		
DADS			0.1		
Total		1001.54	1001.69		
Kcal/kg		4686	4686		

### Measurement of biochemical parameters

Plasma levels of TCand TG were analysed using commercial kits (Asanpharm, Seoul, South Korea). Plasma levels of leptin and insulin were determined using commercial ELISA kits (abcam, Cambridge, UK).

### Western blot analysis

Western blotting was performed as previously described [16]. A bicinchoninic acid assay (#23,225) was used to measure protein concentrations (Pierce, Rockford, IL, USA). Equal amounts of protein extracts 40 µg from liver were separated by a 12% SDS-PAGE and transferred onto an immobile PVDF membrane (BIO-RAD Laboratories, Inc., Hercules, CA, USA) with transfer buffer (25 mM Tris-HCl [pH 8.9], 192 mM glycine, and 20% methanol). The membranes were cut according to molecular weight range of antibodies and incubated with primary antibodies against p-eIF2α (#3398), α, eIF2α(#2103), XBP1(#27,901), CHOP(#2895) from Cell Signalling (Danvers, MA, USA) at 1:1000, and β-actin(A5441) from Sigma-Aldrich (St. Louis, MO, USA) at 1:10000 dilution overnight at 4 °C. The membranes were washed three times, incubated with secondary anti-mouse or anti-rabbit IgG antibodies at 1:1000 dilution, and visualized using enhanced chemiluminescence (SYNGENE, Frederick, MD, USA). The relative protein presence of p-eIF2α, eIF2, XBP1 and CHOP were calculated based on the ratio of intensity of each protein bands to the corresponding β-actin. Band densities were quantified using a ImageJ Launcher .

### Histology and immunohistochemistry

Aortic root tissue were fixed with 10% formalin and dehydrated through an ascending series of alcohols and cleared in xylene using an automated tissue processor (Leica TP 1020, Nussloch, Germany), then embedded in paraffin blocks (*n* = 5). Blocks were cut into 4-µm sections using a rotary microtome, then mounted on glass slides. Sections were deparaffinized with xylene and rehydrated through decreasing concentrations of ethanol. Sections were stained with hematoxylin and eosin (H&E) according to Azemi AK et al. [17], and histologically analyzed using an Olympus microscope (SV40; Olympus, Tokyo, Japan) at 200× magnification.

Paraffin-embedded sections were deparaffinized for immunohistochemical analysis. Aortic sections were treated with 1% H_2_O_2_ and blocked with 5% skim milk in PBS. They were then incubated with mouse primary anti-GRP78 antibody and stained using an avidin-biotin complex kit (Vector Laboratories, Burlingame, CA, USA). Immunostaining was performed using a 3,3ʹ-diaminobenzidine kit (Vector Laboratories). Sections incubated with 10% non-immune mouse serum were used as negative controls.

### Statistical analysis

The two-tailed, unpaired Student’s *t*-test used to test the significance of intergroup differences. Statistical analysis was performed using R software and Prism & software. A *p*-value < 0.05 was considered to denote statistical significance unless otherwise indicated.

## Results

### Effects of DADS on body and tissue weight changes

**Fig. 1:**
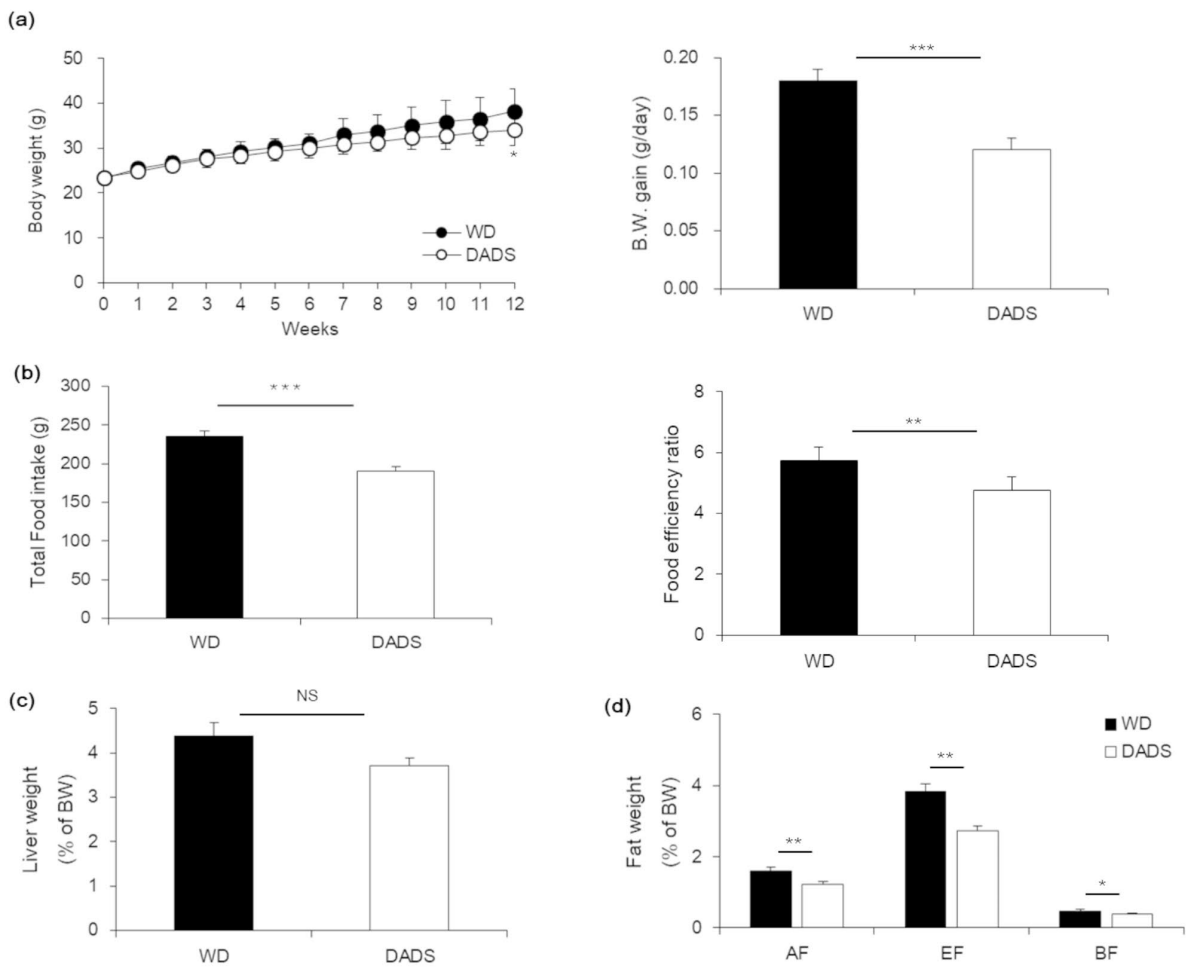
Effects of DADS on body weight and fat weight of ApoE^−/−^ mice fed a Western diet for 12 weeks. **a** Average body weights of mice over time (left panel) and average body weight gain at the end of the experiment (right panel). Black circles indicate Western type diet fed mice, and open circles indicate DADS-supplemented mice. **b** Total food intake of the mice during the experiment (left panel) and average food efficiency ratio of the mice (right panel). The food efficiency ratio was calculated from the body weight gain and food intake. **c** The percent liver weight and **d** fat weight, AF, abdominal WAT; EF, epididymal WAT; BF, brown fat. Black bars indicate the WD group, and open bars indicate the DADS group. The data are presented as the mean ± SE (*n* = 10 per group). *p < 0.05, **p < 0.01, ***p < 0.001, Student’s t-test

As expected, ApoE^−/−^ mice showed increased body weight as well as fat weight on the WD compared to 0.1% DADS treated group, except the liver weight (Fig. 1a, c, d). Compared with that in the WD group, FER was significantly reduced in 0.1% DADS ApoE^−/−^ group (Fig. 1b, *p* < 0.01). Thus, ApoE^−/−^ mice supplemented with 0.1% DADS mitigated body weight gain as well as liver, abdominal, epididymal and brown fat weights increases. These parameters were reduced by 33%, 15%, 25%, 29%, and 18%, respectively, compared with those of the mice in the WD group (Fig. 1d, *p* < 0.01, 0.05).

### Effects of DADS on plasma lipid profiles

**Fig. 2:**
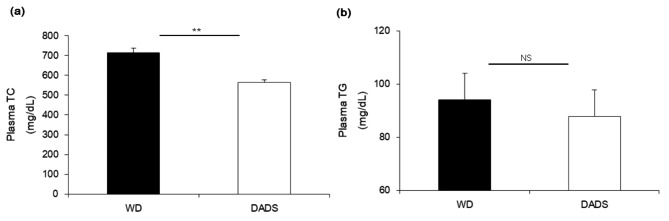
Effects of DADS on plasma TC (**a**) and TG (**b**) levels of ApoE^−/−^ mice fed a Western diet for 12 weeks. Results are presented as the mean ± SE (*n* = 10 per group). **p < 0.01, Student’s t-test

Compared with those of the WD group, levels of plasma TC in ApoE^−/−^ mice supplemented with 0.1% DADS were lower by 21% (714.25 ± 94.14 mg/dL vs. 564.90 ± 87.76 mg/dL, *p* < 0.01). Plasma TG levels did not differ between the WD ApoE^−/−^ and WD + 0.1% DADS ApoE^−/−^ groups (Fig. 2).

### Effects of DADS on plasma leptin and insulin levels

**Fig. 3:**
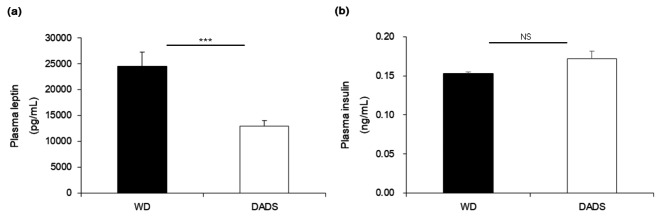
Effects of DADS on plasma leptin (**a**) and insulin (**b**) levels of ApoE^−/−^ mice fed a Western diet for 12 weeks. Results are presented as the mean ± SE (*n* = 10 per group). ***p < 0.01, Student’s t-test

The DADS-supplemented group reduced by 48% plasma leptin levels compared with that in the WD group (*p* < 0.001). Plasma insulin levels were slightly increased by long-term DADS treatment but were similar to those of ApoE^−/−^ mice fed a WD group (Fig. 3). Therefore, in the present study, body weight gain, white fat, and brown fat weights were reduced concomitantly with decreased plasma leptin levels in ApoE^−/−^ mice fed with a DADS-supplemented WD, indicating that DADS may exert a positive effect on body weight gain, FER, fat mass, and WATs weight through regulation of plasma leptin.

### Effects of DADS on the hepatic ER stress markers

**Fig. 4:**
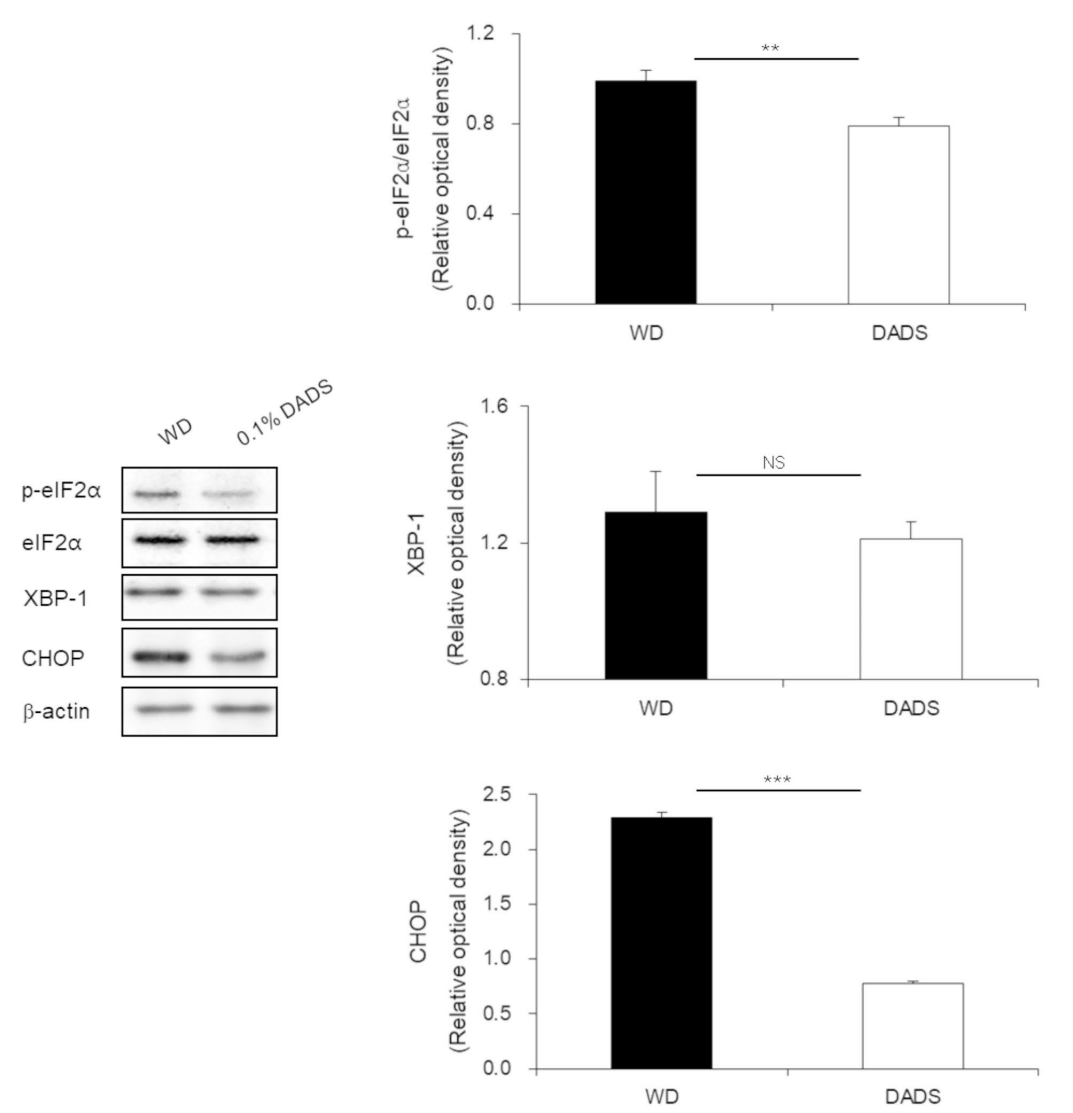
Effects of DADS on endoplasmic reticulum in the liver of ApoE^−/−^ mice fed a Western diet for 12 weeks. Representative protein levels of p-eIF2α, eIF2α, XBP1, and CHOP were measured by Western blotting. Results are presented as the mean ± SE (*n* = 4 ~ 5 per group). **p < 0.01, ***p < 0.001, Student’s t-test

To examine whether DADS inhibits hepatic ER stress, we investigated protein levels in diet-induced hypercholesterolemia models. ER stress markers, including p-eIF2α, CHOP, and XBP-1, were increased in the livers of the WD group, indicating activated UPR state (Fig. 4). In contrast, there was a significant decrease in the protein levels of p-eIF2α and CHOP in the livers of DADS-fed ApoE^−/−^ mice compared to those of mice in the WD group (*p* < 0.01, 0.001). Collectively, this suggests that hypercholesterolemia induced by WD leads to hepatic ER stress markers in WD-fed ApoE^−/−^ mice and that these changes could be mitigated by DADS supplementation.

### Effects of DADS on aortic histopathology

**Fig. 5:**
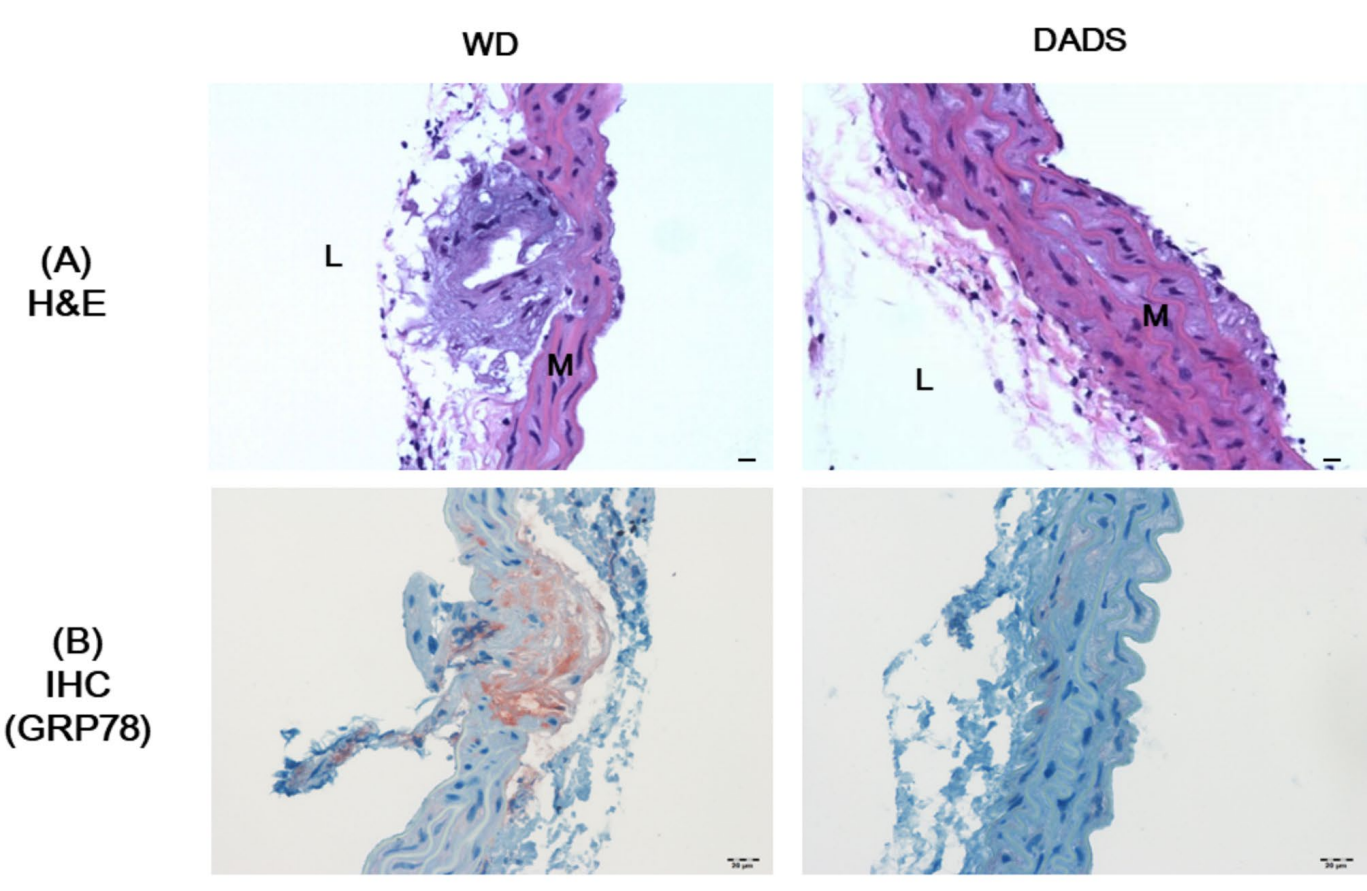
Effects of DADS on histology and immunohistochemistry in the aortic root. Representative Hematoxylin and eosin **(A)** staining (scale bar, 20 μm) and GRP78 immunostaining **(B)** of aortic roots from ApoE^−/−^ mice fed a Western diet for 12 weeks. Original magnification of 200x

H&E and GRP78 immunostaining were performed on aortic root sections to confirm the effect of DADS on histology and level of ER chaperone protein GRP78. Plaques and increased cell proliferation were observed in the WD group but not detected in the 0.1% DADS-supplemented group (Fig. 5a). GRP78 immunostaining revealed increased level in the aortic roots of mice in the WD group compared with that in the WT group (data not shown) In contrast, increased GRP78 level was not observed in the aortas of DADS-supplemented mice (Fig. 5b). These data suggest that hypercholesterolemia-induced ER stress was mitigated by DADS supplementation.

## Discussion

In this study, supplementation with DADS improved leptin resistance and decreased plasma cholesterol, suggesting that DADS intake may improve hyperleptinemia and hypercholesterolemia in WD-fed ApoE^−/−^ mice. Leptin is a fat-derived hormone that controls appetite and body weight by binding to leptin receptors in hypothalamic neurons [18, 19]. Dysfunction of the ER is implicated in the progress of leptin resistance, and induction of the UPR causes inhibition of the leptin signaling network [7]. Pungent food components (i.e. sulforaphane, allicin, capsaicin, and gingerol) affect energy and glucose metabolism and may be involved in the activation of transient receptor potential vanilloid subtype 1 (TRPV1), which is a regulator of leptin signaling [20]. In line with our study, this is accompanied by a reduction in BAT mass and plasma leptin levels in obesity [14, 21]. Furthermore, garlic oil containing diallyl sulfide (DAS), DADS, and diallyl trisulfide (DATS) prevents obesity by increasing energy expenditure and fat oxidation, increasing thermogenesis [13–15, 22]. Interestingly, DAS, DADS, and DATS act as agonists of TRPA1 and TRPV1 [23].

The ER is a crucial organelle for lipid homeostasis, regulating lipid synthesis, assembly, and droplet formation, and ER homeostasis is compromised by the lipid accumulation, leading to ER stress [24–26]. Administration of DADS and DATS attenuates dysfunctional lipid metabolism and inflammatory responses by suppressing the expression of genes involved in hepatic lipogenesis and increasing antioxidant activity in diet-induced obesity conditions [27, 28]. However, DADS also accelerates fatty liver disease by increasing the expression of genes related to lipid biosynthesis in HFD-fed mice [29]. Allicin can relieve hepatic steatosis and maintain energy homeostasis by enhancing mitochondrial β-oxidation and biogenesis, BAT energy expenditure, and oxygen consumption [30]. Our data supports this, showing that the decrease in adipose tissue weight and hepatic ER stress markers by DADS supplementation resulted in a clear improvement of hyperleptinemia and hypercholesterolemia in WD-fed ApoE^−/−^ mice. The accumulation of FC and oxLDLs in infiltrating macrophages within atheroma lesions triggers ER stress-induced apoptosis, which is mediated by CHOP and can be mitigated by antioxidants [31]. These results agree with the results of other studies revealing that 7-ketocholesterol leads to cell death via the IRE1 signaling pathway and dysregulation of calcium homeostasis [6, 32]. S-allyl-L-cysteine, the main bioactive component of garlic, alleviates hepatocyte apoptosis by mitigating the expression of eIF2α and CHOP [33]. In addition, the anticancer effects of organosulfur compounds from garlic are at least partly attributed to increased ER stress, which induces autophagy [34]. In our previous study, 7-ketocholesterol induced a conspicuous increase in ER stress markers and apoptosis [2]. In line with our findings, previous evidence has shown that oxLDL and lipid peroxidation products trigger ER stress and the production of UPR, characterized by GRP78 localization in ApoE^−/−^ mouse vascular cells and atherosclerotic lesions [5, 6, 35, 36], and in the plasma of patients with metabolic disorders [37]. Furthermore, Zhou et al. reported the presence of ER stress inducers (i.e. FC and peroxynitrite) and ER stress markers including GRP78, calreticulin, and CHOP in early stage atherosclerotic lesions [5]. GRP78 mitigates hepatic steatosis by inhibiting insulin- and ER stress-induced genes involved in lipid biosynthesis in obese mice [38]. In addition, GRP78 overexpression inhibited the progression of atherosclerosis by inhibiting apoptosis, thrombin generation, and cholesterol biosynthesis pathways [39]. DATS alleviates mitochondrial apoptosis induced by ER stress via upregulation of GRP78 and CHOP in human carcinoma cells [40]. On the other hand, garlic and garlic-derived sulfur-containing compound were not affect plasma lipid levels and LDL oxidation in mice fed a Western diet, indicating that cardio-protective effect of garlic are not associated with modulation of plasma lipid levels [41].

## Conclusions

Our results demonstrated that dietary supplementation with DADS can significantly alleviate hypercholesterolemia and hyperleptinemia, which is accompanied by a decrease in adipose tissue weight in WD-fed ApoE^−/−^ mice. Moreover, we showed that dietary DADS reduced the severity of atherosclerotic lesions in diet-induced hypercholesterolemia models by inhibiting ER stress, eIF2α, CHOP, and GRP78. Our results suggest that DADS may be a useful food ingredients for preventing diet-induced hypercholesterolemia and cardiovascular diseases.

## Electronic supplementary material

Below is the link to the electronic supplementary material.

**Additional file 1**

## Data Availability

All data generated and analyzed during this study are included in this published article.

## List of abbreviations

ApoE^−/−^: Apolipoprotein E-deficient
BAT: Brown adipose tissue
CHOP: C/EBP homologous protein
DADS: Diallyl disulfide
DAS: Diallyl sulfide
DATS: Diallyl trisulfide
ELISA: Enzyme-linked immunosorbent assay
ER: Endoplasmic reticulum
FC: Free cholesterol
FER: Food efficiency ratio
GRP78/BiP: 78-kD glucose-regulated/binding immunoglobulin protein
H&E: Hematoxylin and eosin
HFD: High-fat diet
oxLDL: Oxidized low-density lipoproteins
TC: Total cholesterol
TG: Tryglyceride
TRPA1: Transient receptor potential cation channel, subfamily A, member 1
TRPV1: Transient receptor potential vanilloid subtyle 1
UPR: Unfolded protein response
WD: Western diet
WT: Wild-type
XBP: X-box binding protein
