# Supplementary figures and images for: Diallyl disulfide alleviates hypercholesterolemia induced by a western diet by suppressing endoplasmic reticulum stress in apolipoprotein E-deficient mice
Source: BMC Complement Med Ther. 2023 May 3;23:141. doi: 10.1186/s12906-023-03920-1 (PMC10155326; doi:10.1186/s12906-023-03920-1)

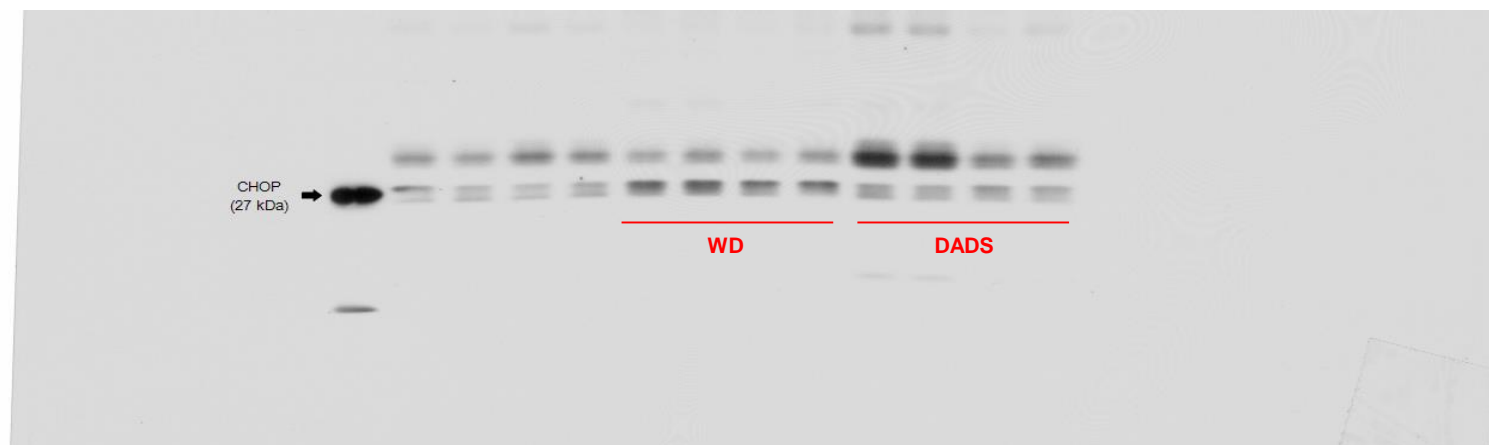

P-eIF2 $\alpha$ (38 kDa)

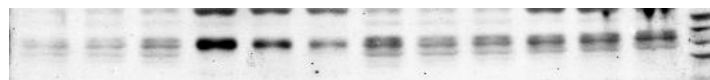

WD

DADS

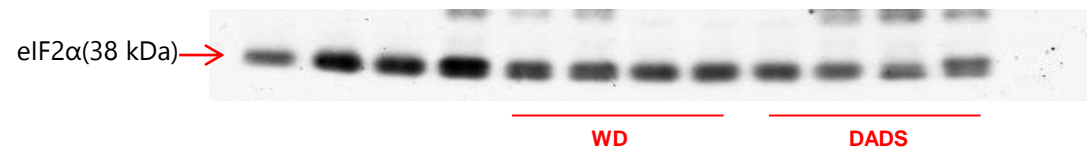

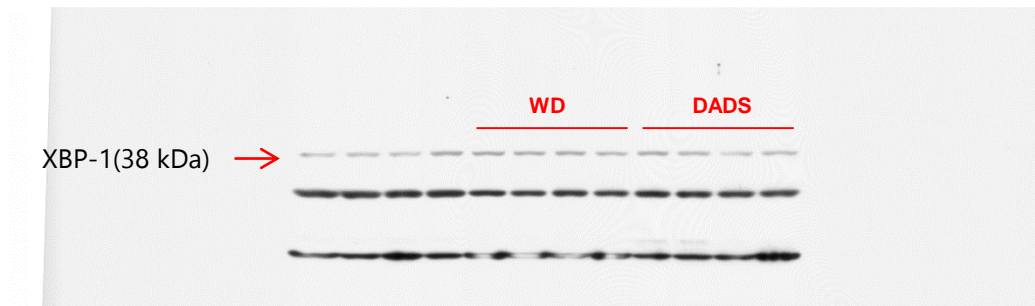

$\beta$ -actin(37 kDa)

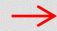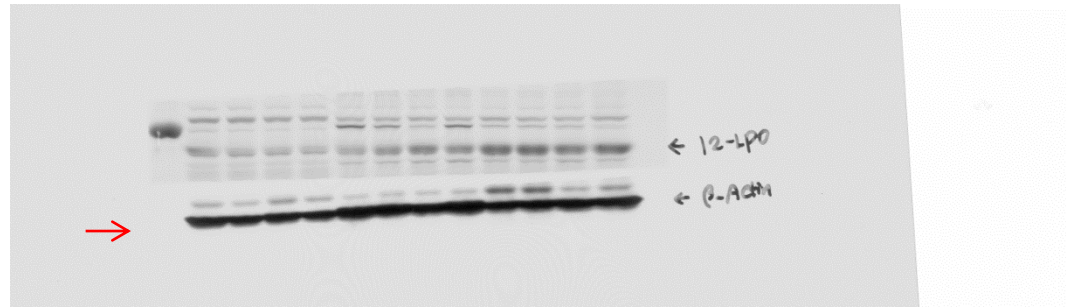

$\beta$ -actin(37 kDa)

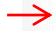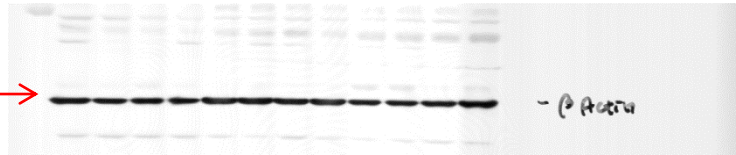

-  $\beta$  Actin

$\beta$ -actin(37 kDa)

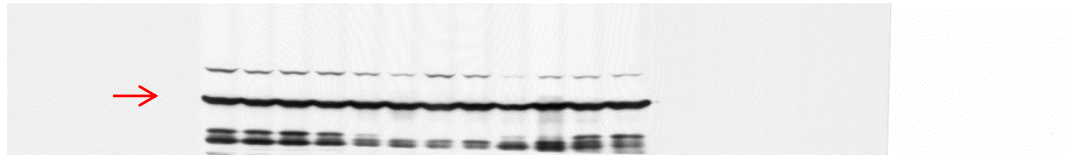

Supplement: Supplementary file 1 — Additional file 1 [file 12906_2023_3920_MOESM1_ESM.pdf]
